# Maximising the Size of Non-Redundant Protein Datasets Using Graph Theory

**DOI:** 10.1371/journal.pone.0055484

**Published:** 2013-02-05

**Authors:** Simon C. Bull, Mark R. Muldoon, Andrew J. Doig

**Affiliations:** 1 Manchester Institute of Biotechnology, Faculty of Life Sciences, The University of Manchester, Manchester, United Kingdom; 2 School of Mathematics, Alan Turing Building, University of Manchester, Manchester, United Kingdom; University of Rome, Italy

## Abstract

Analysis of protein data sets often requires prior removal of redundancy, so that data is not biased by containing similar proteins. This is usually achieved by pairwise comparison of sequences, followed by purging so that no two pairs have similarities above a chosen threshold. From a starting set, such as the PDB or a genome, one should remove as few sequences as possible, to give the largest possible non-redundant set for subsequent analysis. Protein redundancy can be represented as a graph, with proteins as nodes connected by undirected edges, if they have a pairwise similarity above the chosen threshold. The problem is then equivalent to finding the maximum independent set (MIS), where as few nodes are removed as possible to remove all edges. We tested seven MIS algorithms, three of which are new. We applied the methods to the PDB, subsets of the PDB, various genomes and the BHOLSIB benchmark datasets. For PDB subsets of up to 1000 proteins, we could compare to the exact MIS, found by the Cliquer algorithm. The best algorithm was the new method, Leaf. This works by adding clique members that have no edges to nodes outside the clique to the MIS, starting with the smallest cliques. For PDB subsets of up to 1000 members, it usually finds the MIS and is fast enough to apply to data sets of tens of thousands of proteins. Leaf gives sets that are around 10% larger than the commonly used PISCES algorithm, that are of identical quality. We therefore suggest that Leaf should be the method of choice for generating non-redundant protein data sets, though it is ineffective on dense graphs, such as the BHOLSIB benchmarks. The Leaf algorithm is available at: https://github.com/SimonCB765/Leaf, and sets from genomes and the PDB are available at: http://www.bioinf.manchester.ac.uk/leaf/.

## Introduction

Redundancy in datasets of proteins can be defined as the presence of too similar proteins. Redundancy is a barrier to the effective use of the dataset for multiple reasons, most simply size. Redundant sequences in a dataset can prove detrimental to the discovery of novel relations between the proteins, as the presence of similar proteins can bias any conclusions drawn from using that set. Machine learning classifiers trained on redundant training sets will tend to over-fit and be of less value when applied to novel data. A pre-processing step is therefore often used to generate a non-redundant dataset consisting solely of representative proteins from the original redundant set.

Algorithms for determining similarity between proteins are more useful if they work by comparing sequences, rather than structures, since structures are unavailable for most proteins and evolutionary relationships are difficult to quantify. Alignment based approaches to calculating sequence identity are either global or local methods, with local more sensitive when the two sequences may only share isolated regions of similarity, or when scanning a protein database with little to no *a priori* knowledge about the similarity between the database sequences and the query sequence [1]. The predominant heuristics for finding local alignments in proteins are BLAST [2] or PSI-BLAST, which is more sensitive to weak sequence similarities in many cases [3]. BLAST is used by the protein redundancy removal application BlastCuller [4], while PISCES [5] makes use of PSI-BLAST to calculate the pairwise sequence identities.

Our intention here is to maximise the size of the non-redundant dataset. We test both novel and previously published methods that use graph theory. We show that it is possible to use novel graph theoretic methods to increase the size of non-redundant sets, while maintaining identical quality criteria for inclusion of proteins within the set. We find that our novel method, Leaf, generates the largest sets. We apply Leaf to generate non-redundant sets from the PDB, using various sequence similarity and structure quality parameters, and several genomes. Our webpage gives these sets, as well as a facility for users to generate their own non-redundant sets using Leaf.

## Methods

### Solving the Problem of Redundancy through Graph Theory

Sequence similarity relationships between proteins can be shown as a graph: A protein similarity graph *G(V, E)* denotes an undirected graph with vertices *V = {1, 2, … n}* and edges 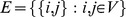. Each protein in the redundant dataset is represented by a vertex. There is an edge between vertices *i* and *j* if the sequence identity of the proteins that *i* and *j* represent is greater than the similarity threshold, here taken to be an upper limit for acceptable mean percentage sequence identity. If the vertex which represents a protein has no edges incident to it, the pairwise sequence identity between that protein and every other protein in the dataset is below the similarity threshold. By representing the dataset and sequence similarities as a graph, it is possible to utilise graph theory to help optimise the generation of the non-redundant dataset.

A non-redundant dataset can be represented by a protein similarity graph that contains no edges. A non-redundant dataset can therefore be generated by removing vertices, and all edges incident to them, from the protein similarity graph until there are no edges remaining. The proteins that correspond to the vertices remaining in the graph will be the non-redundant dataset.

Our goal is to remove nodes, and incident edges, in such a way that the remaining vertices constitute the largest possible set of vertices that have no edges between them. The problem of finding this optimal set is known as the *maximum independent set* (MIS) problem, or equivalently the *stable set* problem. The MIS is the largest possible independent set within a graph. In graph theory, an *independent set* of a graph *G(V,E)* is a set of vertices 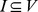 such that 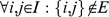. An independent set *I* can be considered to be a *maximal independent set* if the addition of any vertex 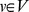 that is not in *I* means that *I* no longer maintains the properties of an independent set. An MIS is a maximal independent set that contains the largest possible number of vertices. Finding the MIS is known to be an NP-complete problem, one where there is no known computationally efficient method for discovering the solution. Approximation based algorithms to find the MIS are thus often used instead.

### Graph Definitions

In order to fully describe the properties of the developed algorithms, definitions of properties of the graphs is necessary: The *neighbourhood* of a vertex *v*, the vertices that share an edge with *v*, in an undirected graph *G(V,E)*, which contains no loops, can be defined as 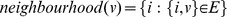. In a protein similarity graph, the neighbourhood of *v* represents all the proteins that have a sequence which is too similar to the protein that *v* represents. The neighbourhood can also be defined for a set of vertices. If *s* is a set of vertices from *G*, then 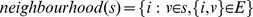. The *degree* of a vertex *v* in *G* can be defined as 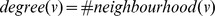. The *support* of a vertex *v* in *G* can be defined as 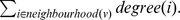


A *clique* 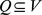 is a subset of the vertices of *G* such that 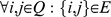. A *maximum clique* of *G* is a largest possible subset of the vertices in *G* for which the clique property is satisfied. A *vertex cover* 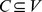 of *G* is a subset of vertices such that every edge in *G* is incident to at least one vertex in *C*. A *minimum vertex cover* of a graph *G* is a vertex cover *C* with the smallest possible number of vertices in it. Graph *components* are sub-graphs that are not connected to each other. Finally, the *complement* of a graph *G(V,E)* is a second graph *H(V,E’)* with the same vertex set, but a complementary edge set. That is, two vertices *i* and *j* are adjacent in *H* if and only if they are *not* adjacent in *G*. A maximum independent set in *G* is thus a maximum clique in *G*’s complement *H*.

### PISCES

The benchmark for all the algorithms developed and tested here is PISCES, as it is very widely used [5], which superseded the previously widely used PDBselect method [6]. PISCES works by listing proteins in order of length. Redundancy is removed by: finding the protein highest up the list that is not marked as kept or removed, and marking it as being kept. For all proteins that have been determined to be too similar to this protein, mark them as being removed. Once the bottom of the list is reached all proteins that have been marked as being kept will be the non-redundant dataset. By only considering proteins higher up the list for inclusion, i.e. proteins with longer sequences, it is possible to miss the opportunity to increase the size of the non-redundant dataset. The returned set will also be biased to include long sequences. Here we evaluate algorithms that use graph theory to maximise the size of the non-redundant dataset while maintaining identical criteria for inclusion (e.g. no two proteins with more than 20% pairwise sequence identity).

### New Algorithms

Two possible graph representations were used for the new algorithms. The first is an *adjacency matrix*. In this representation, an 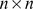 matrix *M* is constructed, where *n* is the number of vertices in the graph. If there is an edge in between two vertices *i* and *j* in the graph, then 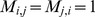. If no edge is present between the two vertices, then 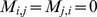. In the *adjacency list* representation, there is one entry in the adjacency list for each vertex in the graph. The list records for each vertex *i* in the graph the vertices in 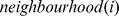. Space is saved over the adjacency matrix representation when the graph is sparse as information is only stored about the presence of edges.

The density of the protein similarity graph of the entire human proteome was calculated for sequence identity thresholds of 20%, 25%, 30%, 40%, 50%, 60%, 70%, 80% and 90%. The highest density was found for the 80% sequence identity threshold, but this was still only 0.03 on a scale where 0 indicates no edges in the graph and 1 indicates that the graph is complete (i.e. all members of G form a single clique). Protein similarity graphs are therefore sparse, since the probability that any two proteins have a high pairwise sequence identity is <3%. An adjacency list representation is therefore utilised.The protein similarity graph is processed before the algorithms are run, by removing all isolated nodes from the graph and adding them to the independent set, as they must all be members of the MIS.

First, we outline three novel algorithms to find an MIS.

### Leaf

The Leaf algorithm works by identifying cliques in the graph that satisfy the criterion of having at least one vertex which is not connected to any vertex outside of the clique. One of the (potentially many) vertices in the clique with no connections outside of the clique is arbitrarily chosen to be kept in the independent set being formed. The algorithm starts by searching for cliques of two vertices which satisfy the criterion. If a clique is found, then one of the vertices in the clique is kept, and the other removed. If no clique is found that satisfies the criterion, then a clique of three vertices is searched for. This process of increasing the number of vertices in the clique being searched for is continued until either a clique is found, or there can be no possible clique in the graph that satisfies the criterion. After a clique has been found and one of its vertices has been incorporated into the growing independent set, the process of searching for a clique begins again with searching for a clique of two vertices. There is no clique in the graph that satisfies the criterion if the size of the clique being searched for is over a certain threshold size. This threshold size is determined dynamically, and is equivalent to the number of neighbours of the highest degree vertex in the graph. Although this upper bound could be tightened through more careful analysis of the graph, searching for a tight upper bound involves finding the size of the maximum clique in the graph. If no clique satisfying the criterion can be found, then the NeighbourCull algorithm is used to determine which vertex to remove. As this method removes the most connected vertex, the upper bound of the size of the clique being searched for will decrease.

An outline of the algorithm is shown below: First the set of vertices that are not in the maximal independent set is initialised (line 1). Next a loop is entered (lines 2–18), which is only exited once there are no edges remaining in the graph (lines 4 and 5). If there are edges remaining, then the next step is to select a vertex to add to the independent set, or one to remove from graph. First the variable *nClique* is initialised (line 6). This is the size of the neighbourhood that all vertices in the clique being searched for must possess. A loop is entered to search for sequentially larger cliques (lines 7–14). If a clique is found where there is at least one vertex *v* in the clique that does not share any edges with a vertex not in the clique, then *v* is to be added to the independent set being formed (lines 8–12). If no clique of a given size is found, then the size of clique being searched for is incremented (lines 13 and 14). If the loop in lines 7–14 terminates without finding a clique, then the NeighbourCull method is used to determine a vertex to delete (lines 15–18).

1. *Removed* 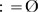

2. While *True*

1. *max* 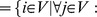 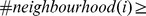 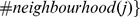

2.  If #*neighbourhood*(*max*) = 0
3.   Return *Removed*
4. *nClique* 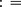
1
5.  While 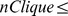 #*neighbourhood*(*max*)
6.   If there is a clique *C* of *nClique* +1 vertices that satisfies the criterion for Leaf
7. *toKeep 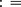
i* where 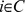
that satisfies the criterion for Leaf
8. *Removed* 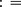 *Removed* 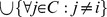

9. <Update the adjacency list to reflect the removal of vertices in *C* that are not *i*>
10.   Exit the inner while loop
11.  Else
12. *nClique* 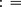 *nClique* +1
13.  If *nClique*>#*neighbourhood*(*max*)
14.   Use NeighbourCull to determine the vertex *v* to remove
15. *Removed 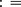
Removed* 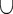 *v*
16. <Update the adjacency list to reflect the removal of *v*>

### NeighbourCull

The NeighbourCull algorithm is based on the goal of removing a vertex which has the highest connectivity (i.e. the most neighbours), but is minimally connected to the vertices not in its neighbourhood. The algorithm works by identifying the vertices with the most neighbours. If there is only one vertex with the most neighbours, then this vertex is removed. When multiple vertices have the most neighbours, the tie is broken by examining the neighbours of the neighbours of the original vertex (i.e. all vertices reachable by traversing two edges). The set of vertices reachable by traversing two edges is determined, and the vertex with the smallest set of vertices reachable in this manner is removed. If there are still multiple vertices which cannot be decided between, then the vertex to remove is chosen arbitrarily from amongst the remaining possibilities.

An outline of the procedure is sketched below: First the set of vertices that are not in the maximal independent set is initialised (line 1). Following this, the loop (lines 2–14) that determines which vertices to exclude from the maximal independent set is entered. The first step is to find those vertices in the graph that still have neighbours (line 3). If there are no vertices with neighbours, and hence no edges in the protein similarity graph, a maximal independent set has been found and the algorithm can exit (lines 4 and 5). If some edges remain, then we find those vertices that have the most neighbours (line 7). If there is only a single vertex that has the maximum number of neighbours, it is marked as not being in the maximal independent set (lines 8–10). However, in cases where more than one vertex has maximal degree, the one to remove is determined by two applications of the *neighbourhood* function (lines 12–14). We compute the size of the *extended neighbourhood*

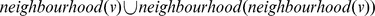



and remove a vertex whose extended neighbourhood is smallest, resolving any remaining ties by an arbitrary choice.

1.  
*Removed* 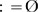

2.  While *True*
 

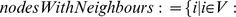
 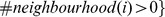

4.   If *nodesWithNeighbours = a*
5.    Return *Removed*
6.   Else
7.  
*max* 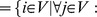 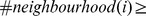 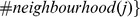

8.    If #*max* = 1
9.  
*Removed* 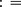 *Removed* 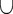 *max*
10. <Update the adjacency list to reflect the removal of *max*>
11.  Else
12. <Select *n 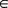
max* such that *n* has the smallest extended neighbourhood>
13. *Removed 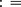
Removed 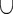 n*
14. <Update the adjacency list to reflect the removal of *n*>

### FIS

The third new algorithm works by first initialising a maximal independent set, and then permuting it in an attempt to increase its size. The algorithm’s first step is to determine the initial vertex from which the maximal independent set will be generated. This is the vertex with the fewest neighbours, with ties broken arbitrarily. From this initial vertex, the set is permuted using the *addnodes* sub-function. This takes as its arguments the current independent set, and the set of all the vertices in the graph. This function works by first determining if there are any vertices that are not adjacent to the current independent set. If there are no non-adjacent vertices, then the current independent set is returned. If there are vertices which are not adjacent to the current independent set, then the independent set can be extended by adding a new vertex. This is done by finding the non-adjacent vertex which, when added to the independent set, causes the fewest vertices that are currently not adjacent to the independent set to become adjacent. The function *swapnodes* is used to see if the size of the independent set can be increased by making small alterations to the vertices in the set. The vertices that are not in the independent set are tested one at a time to see how many vertices from the independent set they are adjacent to. If a vertex *i* that is not in the independent set is adjacent to only one vertex *j* that is, then *i* and *j* can be swapped without invalidating the properties of a maximal independent set. The new independent set resulting from this swap is passed to *addnodes* to see if it can be extended by the addition of any non-adjacent vertices.

An outline of the algorithm, including its two sub-functions *addnodes* and *swapnodes*, is shown below: The algorithm’s first step is to determine the initial vertex from which the maximal independent set will be generated. This is done in line 1, and is chosen to be the vertex with the fewest neighbours, with ties broken arbitrarily. From this initial vertex a maximal independent set is generated (line 4), and following this the set is permuted in an attempt to increase its size (line 5). Once the set has been permuted, either the permuted independent set (line 7) or the non-permuted set (line 9) is returned based on which contains a greater number of vertices.

The majority of the work in the algorithm is done in the *addnodes* sub-function. This takes as its arguments the current independent set, and the set of all the vertices in the graph. This function works by first determining if there are any vertices that are not adjacent to the current independent set (line 12). If there are no non-adjacent vertices, then the current independent set is returned (lines 13 and 14). If there are vertices which are not adjacent to the independent set being formed, then the independent set can be extended by adding a new vertex (lines 15–19). This is done by finding the non-adjacent vertex which, when added to the independent set, causes the fewest vertices that are currently not adjacent to the independent set to become adjacent. The number of currently non-adjacent vertices that will become adjacent if a vertex *i* is added to the independent set *Ind* is determined to be 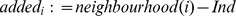. Therefore the vertex *j* that is added to *Ind* is chosen such that 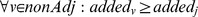, where *nonAdj* is the set of all vertices that are not adjacent to *Ind*.

1.  <Select the vertex *I* such that 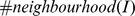 is minimal>
2.  
*Ind* 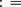
{*I*}
3.  <Set *V* to all the vertices in the graph>
4.  
*Ind_A_*: =  *addnodes* (*Ind*, *V*)
5.  
*Ind_S_*: =  *swapnodes* (*Ind_A_*, *V*)
6.  If 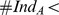 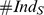

7.   Return 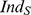

8.  Else
9.  Return 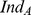

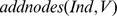
:

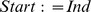

11. While *True*

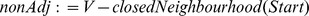

13.  If *nonAdj* = 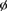

14.   Return *Start*
15. For *i* in *nonAdj*

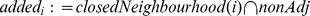

17.  If <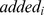 contains the smallest number of vertices for all 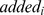
found so far>
18. *min 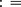
i*
19. *Start* 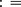 *Start 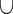 min*

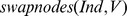
:

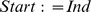
, 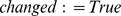, *maxSet
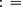
Ind*
21. While *changed*
22.  
*changed
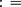
False*
23.  For *i* in 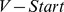

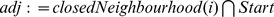

25.   If 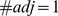

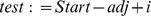

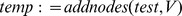

28.    If #*temp*> #*maxSet*
29. *maxSet 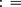
temp*

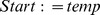

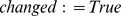

32. Return *maxSet*

The function *swapnodes* is used at the end of the algorithm to see if the size of the independent set generated by line 4 can be increased by making small alterations to the vertices in the set. The vertices that are not in the independent set are tested one at a time to see how many vertices from the independent set they are adjacent to (lines 23–31). If a vertex *i* that is not in the independent set is adjacent to only one vertex *j* that is, then *i* and *j* can safely be swapped without invalidating the properties of a maximal independent set (line 26). The new independent set resulting from this swap is passed to *addnodes* to see if it can be extended by the addition of any non-adjacent vertices (line 27). If the set returned by *addnodes* contains more vertices than the largest maximal independent set previously found it is recorded as the current best maximal independent set (lines 28–31).

### Examples

**Figure 1:**
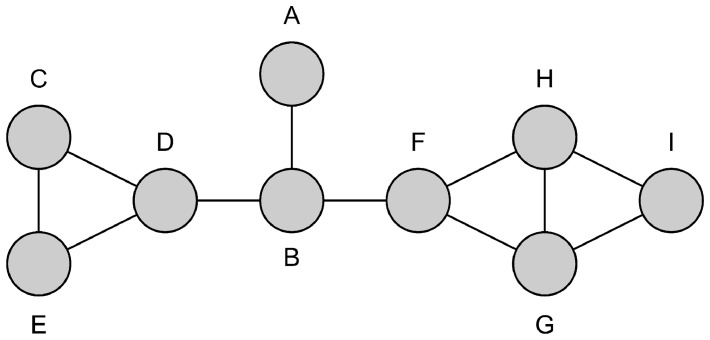
An example graph to demonstrate the differences between Leaf, NeighbourCull and FIS.

The simplest method of fully understanding the new algorithms is through an example which demonstrates the differences between them. The graph in Figure 1 is one such graph, and will be used to illustrate the execution of the Leaf, NeighbourCull and FIS algorithms. For all three algorithms the alphabetic names of the vertices will be used to arbitrarily break any ties.

**Figure 2:**
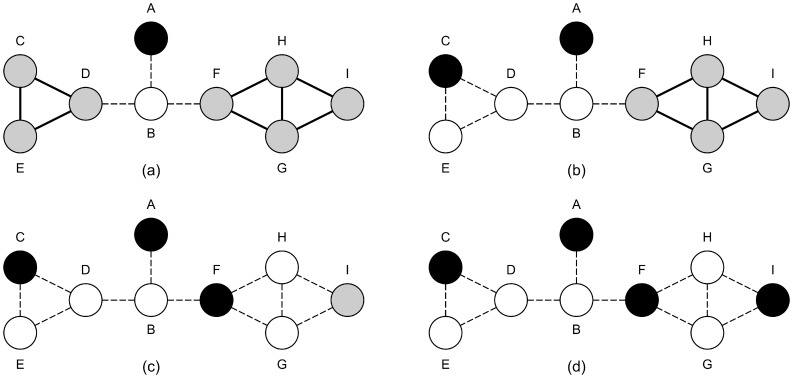
The progress of execution of the Leaf algorithm on the graph seen in Figure 1. Black vertices are in the independent set being generated, white vertices have been removed and grey vertices are those that are still to be decided upon. Dashed edges indicate edges that have been removed from the graph due to a vertex being removed. Each graph corresponds to the results of one the execution steps of the Leaf algorithm. (a) corresponds to step 1, (b) to step 3, (c) to step 5, (d) to step 6.

The execution of the Leaf algorithm on the graph in Figure 1 is as follows:

1. Select vertex *A* to keep. This is because vertices *A* and *B* comprise the only maximal clique of two vertices. Vertex *B* is not kept because it is connected to vertices that are not in the clique (Figure 2).
2. There are no more maximal cliques of two vertices, so cliques of three vertices are examined.
3. Select vertex *C* to keep. There are three maximal cliques of three vertices ({*C,D,E*}, {*F,G,H*}, {*G,H,I*}), all of which contain at least one vertex that has no connection to a vertex not in the clique. Clique {*C,D,E*} is arbitrarily chosen as the one to keep a vertex from. Vertex *C* is chosen arbitrarily from this clique. (Figure 2).
4. There are no maximal cliques of two vertices, so cliques of three vertices are examined.
5. Select vertex *F* to keep. Clique {*F,G,H*} is arbitrarily chosen as the maximal 3-clique to keep a vertex from. Vertex *F* is the only vertex in the clique that has no connections to vertices not in the clique. Therefore vertex *F* is kept. (Figure 2).
6. Keep vertex *I* as it has no neighbours (Figure 2).
7. The final independent set is {*A,C,F,I*}.

**Figure 3:**
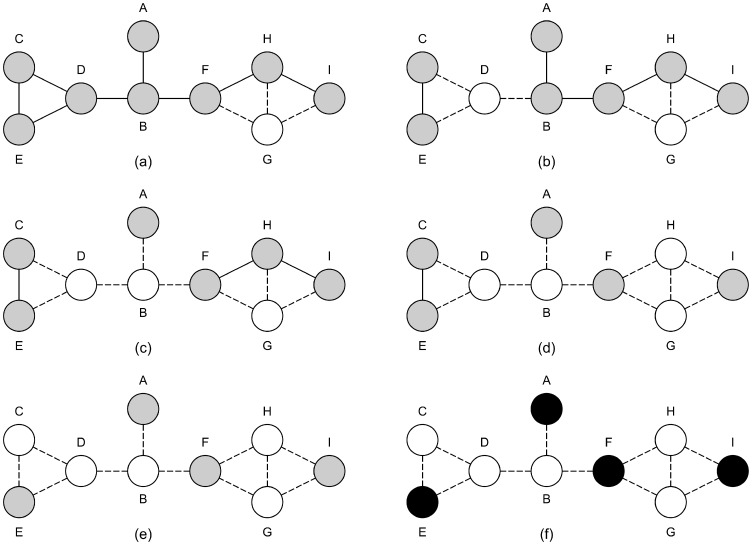
The progress of execution of the NeighbourCull algorithm on the graph seen in Figure 1. Black vertices are in the independent set being generated, white vertices have been removed and grey vertices are those that are still to be decided upon. Dashed edges indicate edges that have been removed from the graph due to a vertex being removed. Each graph corresponds to the results of one the execution steps of the NeighbourCull algorithm. (a) corresponds to step 2, (b) to step 4, (c) to step 6, (d) to step 7, (e) to step 8 and (f) to step 9.

The execution of the NeighbourCull algorithm on the graph in Figure 1 is as follows:

1. Vertices *B,D,F,G* and *H* all have three neighbours, and no other vertex has more, so we need to look at the sizes of their extended neighbourhoods to choose a vertex for deletion. The relevant data are summarised in Table 1 where, in the column headings, 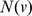 is an abbreviation for *neighbourhood*(*v*) and 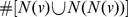 is the size of the extended neighbourhood.
  Vertices *G* and *H* have the smallest extended neighbourhoods, and so vertex *G* is arbitrarily chosen to be removed instead of *H* (Figure 3).
2. Vertices *B* and *D* now have the most neighbours of the remaining vertices, 3, while their extended neighbourhoods contain 7 and 6 vertices, respectively. Thus we remove vertex *D*.
3. Now vertices *B,F* and *H* all have two neighbours apiece, while their extended neighbourhoods contain either 4 (for *B* and *H*) or 5 (for *F*) vertices. We choose, arbitrarily, to remove vertex *B*.
4. Vertex *H* will be removed as it has the most neighbours (Figure 3).
5. Vertices *C* and *E* both have the most neighbours, and the same extended neighbourhood. Remove vertex *C* arbitrarily (Figure 3).
6. The final independent set is {*A,E,F,I*} (Figure 3).

**Table 1:** Vertex Neighbourhoods for NeighbourCull Algorithm Example.

Vertex *v*	*N*(*v*)	*N*(*N*(*v*))	*# N*(*v*)	#[*N*(*v*) 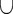 *N*(*N*(*v*))]
B	{*A, D, F*}	{B, C, E, G, H}	3	8
F	{B, G, H}	{A, D, F, G, H, I}	3	7
D	{B, C,E}	{A, C, D, E, F}	3	6
G	{F, H, I}	{B, F, G, H, I}	3	5
H	{F, G, I}	{B, F, G, H, I}	3	5

**Figure 4:**
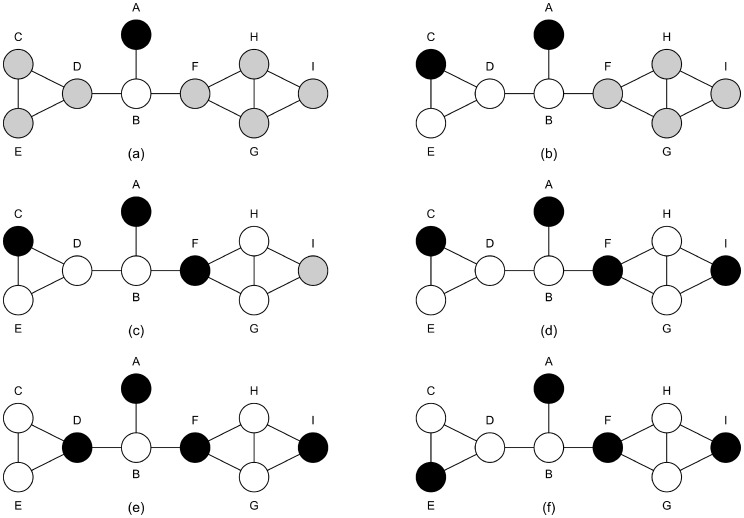
The progress of execution of the FIS algorithm on the graph seen in **Figure 1**. Black vertices are in the independent set being generated, white vertices are the vertices adjacent to the independent set and grey vertices are those that are still to be decided upon. Each graph corresponds to the results of one the execution steps of the FIS algorithm. (a) corresponds to step 1, (b) to step 3, (c) to step 4, (d) to step 5, (e) to step 7 and (f) to step 10.

The execution of the FIS algorithm on the graph in Figure 1 is as follows:

1. The initial vertex is set to *A* as it has the fewest neighbours (Figure 4).
2. *Ind
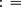
*{*A*}
3. Vertices *C,D,E,F* and *I* would all cause the fewest new vertices, 3, to become adjacent to *Ind.* Vertex *C* is added arbitrarily (Figure 4).
4. Vertices *F* and *I* would both cause the fewest new vertices, 3, to become adjacent to *Ind.* Vertex F is added arbitrarily (Figure 4).
5. Vertex *I* is added to *Ind* as it is the only vertex available to add (Figure 4).
6. *Ind* is {*A,C,F,I*} after the function *addnodes* completes.
7. The first vertex that is not in *Ind*, and is only adjacent to one vertex in *Ind*, is *D*. *D* is swapped with *C*, and addnodes is called with 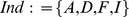 (Figure 4).
8. No additional vertices can be added.
9. The size of *Ind* has not increased so *maxSet* is still {*A,C,F,I*}.
10. The next vertex that is not in *Ind*, and is only adjacent to one vertex in *Ind*, is E. *E* is swapped with *C*, and addnodes is called with 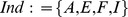 (Figure 4).
11. No additional vertices can be added.
12. The size of *Ind* has not increased so *maxSet* is still {*A,C,F,I*}.
13. No more vertices can be swapped so the final independent set is {*A,C,F,*I}.

### Existing Algorithms

Three algorithms from the literature were chosen to be tested alongside the three new algorithms:

GLP is a state of the art heuristic for approximating the maximum clique that works by finding an initial clique starting from a random initial vertex in the graph, and then improving this initial clique using local search operations [7]. Algorithm 1 from this paper, along with restart rule 2, is used here. An implementation of the algorithm was written in Python. As the GLP algorithm makes use of rules for restarting, it is possible that the algorithm will execute for a substantial length of time on larger graphs. For this reason, a parameter is used with the GLP algorithm that limits the number of vertices that can be added to and removed from the clique being generated. There are two problems with using the algorithm as it stands for the generation of non-redundant datasets. The first is that the size of the maximum clique of the graphs being used was known in the tests done in the GLP paper [7]. This meant that the algorithm could be stopped if the clique being generated ever reached this size. Unfortunately, the protein similarity graphs being used here have unknown MISs, and therefore the algorithm cannot be stopped early in the same way. Secondly, the value of the parameter cannot easily be set to prevent the algorithm running for excessive lengths of time. In order to prevent this, GLP was adapted to allow a time limit to be placed on the running of the algorithm and the largest maximal independent set found up to that point is returned.

The final two algorithms work based on the neighbourhood of the individual vertices in the graph. The VSA algorithm of Balaji *et al*. [8] finds an approximation to the MIS by calculating an approximation to the minimum vertex cover. The algorithm works by beginning with an empty vertex cover, and progressively increasing the number of vertices in the vertex cover by adding the vertex with the highest support. If two vertices have the largest value for the support, then the vertex with the higher number of neighbours is added. This is repeated until there are no vertices that are not either in the vertex cover or adjacent to a vertex in the vertex cover. This algorithm was re-implemented in Python and extended to incorporate a limit on the length of execution.

The final algorithm used was BlastCuller [4]. Unlike GLP and VSA, this algorithm is designed to produce non-redundant protein datasets. BlastCuller generates a non-redundant dataset by approximating the MIS of the protein similarity graph. The algorithm works by initialising the result as an empty set, and then adding to it all the isolated vertices. Following this, the vertex with the most neighbours is deleted, and any newly isolated vertices are added to the result set. This process is repeated until all vertices have been either removed, or added to the result set. BlastCuller was re-implemented in Python for the tests here, in order to enable a time limit on the exaction to be incorporated.

### Cliquer

Although there are no known efficient algorithms to compute the MIS of an arbitrary graph, it is nonetheless possible to find the size of the MIS exactly using so-called branch-and-bound algorithms, which have worst-case running times that are exponential in the number of vertices. These algorithms typically combine a brute-force search (list all possible subsets of the vertex set and ask whether each is an independent set, keeping track of the largest set seen so far) with a clever upper bound that allows one to prove statements such as “any independent set that includes vertices 1, 25, 1548 and 21973 contains at most 53 other vertices” and so eliminate whole families of subsets without having to enumerate and check each member.

To obtain exact answers against which to check our algorithms we used the Cliquer library [9]–[10] to find a maximum clique in the complement of the protein similarity graph. Cliquer works by successively computing the size *c_i_* of the maximum clique in the subgraph that contains only the vertices in the set 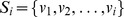
and any edges running between them. It’s clear that either 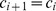 or 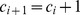, with the latter holding only when there is a maximum clique in 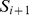 that includes the vertex 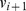: this is the key observation behind the upper bound that speeds Cliquer’s search. The algorithm’s running time depends on the order in which the vertices are listed and we used Cliquer’s default ordering strategy, which proceeds in two stages. Initially the vertices are arranged in order of decreasing degree; one then uses the greedy colouring algorithm recursively to choose large sets of non-adjacent vertices. The final vertex ordering lists the vertices in order of increasing colour-index (as assigned by the greedy colouring stage) and, within each colour-group, in order of decreasing degree.

### Experimental Design

The algorithms were compared in terms of the number of proteins removed from the original redundant dataset of the human proteome (downloaded from http://www.uniprot.org/downloads on December 10th, 2010), and the time taken to finish. Pairwise sequence identities between all possible pairs of the 20251 human proteins were calculated using PSI-BLAST version 2.2.25. PISCES was used to perform the BLASTing, and to process the resulting alignment information. The BLOSUM62 scoring matrix was used. From this alignment file, it was possible to determine which proteins had a percentage pairwise sequence identity over any specified threshold.

Random datasets of 500, 1000, 2000 and 5000 proteins were generated by sampling from the 20251 human proteins downloaded from UniProt. 50 datasets were generated randomly of each size. Taking the 2000 protein datasets as an example, the process for generating datasets was as follows:

1. Select 2000 different proteins from the 20,251 possible proteins.
2. Extract any alignments from the alignment file where the 2000 proteins were either the query or the hit in the PSI-BLAST output.
3. Form an alignment file from the subset of entries selected in step 2, and a FASTA format file of the proteins selected in step 1.
4. Repeat steps 1–3 until 50 datasets have been generated.

This method of generating datasets ensures that the same protein is not present multiple times in any one dataset, but may be present in more than one dataset of any given size.

Individual datasets were tested as follows:

1. Generate an adjacency list for each percentage threshold (20%, 25%, 30%, 40%, 50%, 60%, 70%, 80% and 90%).
2. Run PISCES on the dataset using each of the nine percentage thresholds.
3. Run each of the algorithms being tested on each of the nine adjacency lists.

The time limit for each algorithm was set to be the longer of either two minutes or ten times the running time of the Leaf algorithm, whichever is the longer. We used a PC running Windows XP SP3, with a 3.30 GHz Intel i3–2120 processor and 4Gb of 1600MHz DDR3 RAM.

The model organism data used for the comparison of the algorithms was downloaded from UniProt on November 3rd 2011. For each proteome, only reviewed proteins in the complete proteome were downloaded. The taxonomy ID for the proteomes was: 9606 for *H.sapiens*, 10090 for *M. musculus*, 83333 for *E. coli*, 559292 for *S. cerevisiae* and 3702 for *A. thaliana*.

The algorithms were also tested using a benchmark suite of graphs, BHOSLIB (http://www.nlsde.buaa.edu.cn/~kexu/benchmarks/graph-benchmarks.htm). This benchmarking serves to test the ability of the algorithms to find a MIS in general, rather than simply from protein similarity graphs. Additionally this should give an idea of how the algorithms designed for the simpler protein similarity graphs fare on more complex graphs in comparison to state of the art heuristics.

As of August 23^rd^ 2012, only the PISCES and Leaf algorithms are available online for users to use.

## Results

### Subsets of the Human Proteome

The quality of each algorithm tested is measured as the number of proteins removed from the starting set, where the smaller the number removed, the better (Table 2).

**Table 2:** Mean Number Over 50 Runs Kept from Datasets of 100, 250, 500, 1000, 2000 and 5000 Proteins.

Cut Off	PISCES	Leaf	FIS	NeighbourCull	VSA	BlastCuller
**500 Proteins**						
20%	371.9	384.3	384.1	384.0	380.4	383.4
25%	413.4	419.4	419.3	419.3	417.6	419.0
30%	442.2	445.1	445.0	445.0	444.5	444.8
40%	472.3	474.5	474.5	474.5	474.1	474.4
50%	488.5	489.0	489.0	489.0	488.9	489.0
60%	493.4	493.4	493.4	493.4	493.4	493.4
70%	496.0	496.0	496.0	496.0	496.0	496.0
80%	497.2	497.2	497.2	497.2	497.2	497.2
90%	498.4	498.4	498.4	498.4	498.4	498.4
**1000 Proteins**						
20%	668.0	699.6	698.7	698.0	688.2	695.8
25%	766.9	785.7	785.4	785.3	779.9	784.0
30%	840.2	849.2	849.0	849.0	846.9	848.6
40%	917.4	922.0	921.9	921.9	920.6	921.6
50%	958.4	960.3	960.3	960.3	960.2	960.3
60%	976.6	977.0	977.0	977.0	977.0	977.0
70%	985.5	985.6	985.6	985.6	985.6	985.6
80%	990.4	990.6	990.6	990.6	990.6	990.6
90%	994.8	994.9	994.9	994.9	994.9	994.9
**2000 Proteins**						
20%	1158.2	1240.1	1236.2	1234.9	1210.1	1229.9
25%	1364.8	1421.0	1419.4	1418.4	1402.1	1414.8
30%	1544.7	1575.5	1574.8	1574.6	1566.2	1573.0
40%	1754.6	1768.7	1768.2	1768.4	1765.6	1767.9
50%	1864.1	1870.1	1870.1	1870.1	1869.3	1870.1
60%	1920.1	1922.1	1922.1	1922.1	1922.0	1922.1
70%	1947.9	1948.9	1948.9	1948.9	1948.8	1948.9
80%	1980.6	1980.9	1980.9	1980.9	1980.9	1980.9
90%	1980.6	1981.0	1981.0	1981.0	1981.0	1981.0
**5000 Proteins**						
20%	2284.0	2520.0	2504.7	2503.3	2439.7	2491.8
25%	2777.9	2970.9	2962.7	2959.5	2901.3	2947.7
30%	3293.0	3423.1	3419.2	3417.7	3380.8	3410.7
40%	3997.3	4052.1	4050.5	4050.6	4040.9	4048.5
50%	4385.4	4417.8	4417.8	4417.5	4413.0	4416.9
60%	4622.7	4634.1	4634.0	4634.0	4632.7	4633.7
70%	4756.5	4762.5	4762.5	4762.5	4762.2	4762.4
80%	4905.6	4908.5	4908.5	4908.5	4908.4	4908.5
90%	4905.8	4908.7	4908.7	4908.6	4908.6	4908.7

### GLP

When the execution time of the GLP algorithm was limited to ten times that of the Leaf algorithm, GLP performed more poorly than Leaf and often worse than PISCES (Figure S1). This is mainly due to GLP terminating before it has had a chance to build up a maximal independent set in the protein similarity graph.

**Figure 5:**
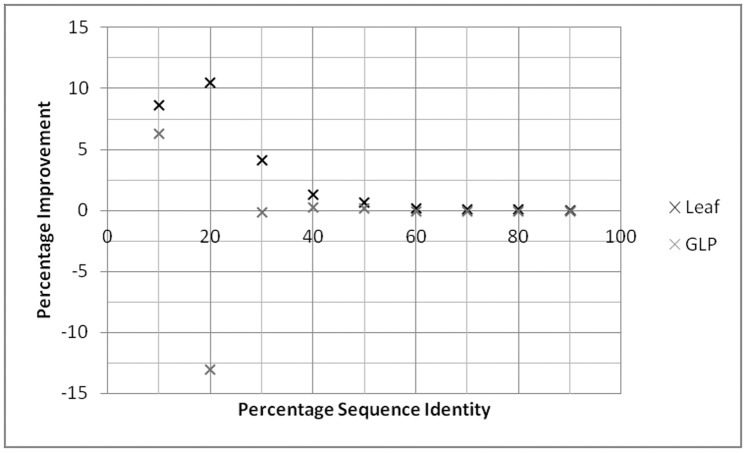
Comparison of Leaf and GLP to PISCES for 5000 protein data sets, when GLP is terminated after executing for 500 times as long as Leaf. The percentage improvement over PISCES is calculated at each cut off percentage as (# proteins kept by algorithm - # proteins kept by PISCES)/(# proteins kept by PISCES) * 100. For example, if the non-redundant dataset found by Leaf is 2000 and for PISCES it is 1900, then the percentage improvement is (2000–1900)/1900 * 100 = 5.26.

In order to determine whether running GLP for a longer length of time will increase the size of the non-redundant dataset generated, we extended the time limit for the execution of GLP to 500 times the Leaf execution time and studied a subset of the 5000 protein datasets (Figure 5). The improvement over PISCES was lower for GLP than for Leaf at all sequence identity thresholds. At all but 40% and 50% sequence identity, the results for GLP were worse than those of PISCES.

### Leaf, FIS, NeighbourCull, VSA and BlastCuller

At sequence identities greater than 50%, there is little improvement over PISCES for any of the algorithms (Table 2). Gains are small at the higher sequence identities because the protein similarity graphs themselves contain only a few proteins. For example, using a 90% sequence identity threshold with datasets of 1000 proteins generates protein similarity graphs with a mean of 9.68 proteins, and the mean number of nodes in each component is 2.68. The small size of the components leaves very little room for an improvement in the size of the non-redundant dataset.

For sequence identity thresholds below 60%, the improvement over PISCES achieved by all five algorithms is more substantial (Table 2). The pattern of improvement changes depending on the sequence identity threshold used. One trend that is noticeable across all sequence identity thresholds is the increasing difference between the five algorithms as the datasets increase in size.

The order of success of the algorithms is the same for almost every combination of dataset size and sequence identity threshold, with Leaf showing the most improvement followed by FIS, NeighbourCull, BlastCuller and finally VSA. The three algorithms that work solely by identifying the vertex that is most connected by some measure show the smallest improvement over PISCES.

Table 3 shows the time taken for the 5000 protein data set at sequence cut offs from 20–90%. We see that all the algorithms apart from GLP take a similar time to PISCES. Leaf usually takes the least time, even though it tends to give the largest sets. The mean lengths of the proteins returned by each algorithm from the human proteome for the 20% cut off are: FIS 419.9; Leaf 410.7; NeighbourCull 422.6; VSA 418.5; BlastCuller 422.6 and PISCES 518.3. Figure S2 shows a cumulative frequency plot. PISCES thus does select for longer chains, as expected.

**Table 3:** Mean Execution Times for 5000 Protein Data Set (s).

Cut Off	PISCES	Leaf	FIS	NeighbourCull	GLP	VSA	Blastculler
20%	3.454	1.944	10.747	3.339	120.868	7.920	2.966
25%	3.390	0.840	5.961	1.998	120.440	3.756	1.472
30%	3.337	0.693	1.713	0.610	120.229	0.931	0.364
40%	3.315	0.089	0.043	0.039	120.009	0.054	0.022
50%	3.288	0.020	0.011	0.016	120.004	0.013	0.008
60%	3.297	0.009	0.003	0.010	120.002	0.005	0.004
70%	3.339	0.005	0.002	0.008	120.001	0.003	0.002
80%	3.361	0.003	0.001	0.004	120.001	0.002	0.002
90%	3.370	0.001	0.001	0.002	120.000	0.001	0.001

### Comparisons to the Maximum Independent Set

The Cliquer algorithm computes the exact size of a maximum independent set, which is the perfect solution to our problem of finding the largest possible non-redundant protein data set. Unfortunately, it is so slow that it is only possible to find the MIS for starting sets of 1000 proteins or fewer with this method. We ran starting sets of 5000 proteins and none reached a solution after 6 months of processing on a Condor [11] distributed computing pool. Jobs submitted to this pool run mainly on inactive, recent-model desktop machines in student computing clusters and, during the academic term, get around 8–10 hours of uninterrupted processor time per day. Nevertheless, we can compare the approximate methods used here to the exact solution for sets of 500 and 1000 proteins. Table 4 shows these comparisons. For the 1000 proteins subsets, Leaf misses the MIS in only a few cases, shown, for example, by the mean difference for the 20% cut off being only 0.1 proteins. This gives reassurance that we have found highly accurate algorithms that can reach, or get close to, the MIS in a short time. For example, with the 1000 protein sets at a 20% cut off, the Cliquer algorithm takes on average 4130 seconds to find the MIS for each set, while the Leaf method needs only 42ms and nearly always finds the MIS.

**Table 4:** Number of Proteins Present in the exact Maximum Independent Set with Differences to the MIS for each Algorithm.

500 Proteins								
Cut Off	Exact Kept	NeighbourCull	FIS	Leaf	VSA	BlastCuller	GLP	PISCES
20%	385.34	0.3	0.3	0.07	4.0	1.00	4.7	11.5
30%	445.9	0.2	0.1	0	0.6	0.4	0.2	2.7
40%	476.1	0	0	0	0.4	0.07	0.7	2.3
50%	489.9	0	0	0	0.07	0	0.3	0.3
60%	494.0	0	0	0	0	0	0	0.03
70%	496.2	0	0	0	0	0	0	0
80%	497.3	0	0	0	0	0	0	0
90%	498.4	0	0	0	0	0	0	0
**1000 Proteins**								
20%	699.7	1.7	1.0	0.1	11.5	3.9	40.3	31.7
30%	849.2	0.2	0.2	0	2.3	0.6	174	9.0
40%	922.0	0.2	0.2	0.06	1.4	0.4	2.4	4.6
50%	960.3	0.02	0	0	0.2	0.02	1.2	1.9
60%	977.0	0	0	0	0	0	0	0.4
70%	985.6	0	0	0	0	0	0	0.2
80%	990.6	0	0	0	0	0	0	0.1
90%	994.9	0	0	0	0	0	0	0.1

### Human Proteome

The results of running the algorithms on the entire human proteome (20251 proteins) are in Table 5. Leaf again outperformed the other algorithms in most cases.

**Table 5:** Number of Proteins Kept from Human Proteome.

Cut Off	PISCES	Leaf	FIS	NeighbourCull	VSA	BlastCuller
20%	5700	6643	6572	6580	6365	6541
30%	9007	9856	9796	9800	9594	9762
40%	12422	12843	12832	12829	12746	12811
50%	14927	15169	15167	15164	15129	15154
60%	16771	16887	16884	16886	16874	16884
70%	17969	18036	18036	18036	18030	18033
80%	18763	18801	18801	18801	18798	18801
90%	19366	19389	19388	19389	19388	19388

GLP was originally used in the test on this dataset, but the size of the representation of the protein similarity graph proved to be problematic. For example, the largest connected component of the protein similarity graph at 20% sequence identity contains 16,383 vertices, and has a mean degree of 84. The complement of this graph will therefore have the same number of vertices, but a mean degree of 16,299. In order to record all the connections between vertices in the graph, approximately 266 million connections need to be recorded. The size of the graph representation will cause the algorithm to be substantially slower, making the time required to generate a non-redundant dataset prohibitive. Similar issues may explain the poor results of GLP on the random subsets of the human proteome.

### Model Organisms

We applied MIS algorithms to the *M. musculus, E. coli, A. thalania* and *S. cerevisiae* proteomes, in order to evaluate its performance on diverse proteomes and to generate potentially useful data sets for groups studying these organisms. Table S1 summarises their performances and shows that again Leaf consistently gives the largest culled sets.

### PDB

Non-redundant sets of protein crystal structures are often used to study protein structure. PDB files can be culled not just on the maximum pairwise sequence identity, but also structure quality, as measured by minimum resolution and R-factor. We used the Leaf algorithm to compare with PISCES, using a range of these parameters (Table S2). For sets with low sequence identities (20% –25%), Leaf returns data sets that are around 10% larger than from PISCES.

### BHOSLIB Benchmark

**Figure 6:**
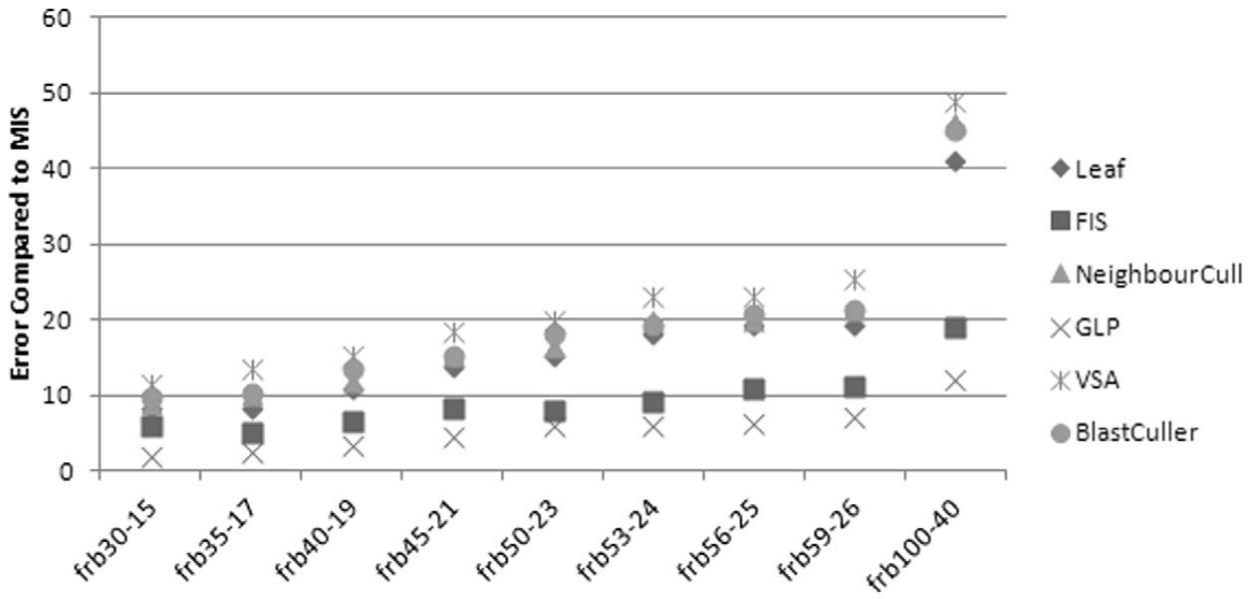
Algorithm comparison for the BHOSLIB Benchmarks. BHOSLIB datasets are listed as, for example, 30–15, where the datasets consist of 30 cliques of 15 nodes each (450 nodes in total) and the MIS is 30 nodes in size.

The results of running Leaf, FIS, GLP, VSA and BlastCuller on the BHOLSIB benchmark datasets can be seen in Figure 6, where the mean difference between the number of vertices returned by the algorithms and the true MIS is shown.

GLP consistently outperforms the other algorithms on the benchmark datasets, unlike the protein datasets. For the other algorithms, the structure of the graphs is not suitable for the simple methods used to generate the independent sets. For example, the Leaf method relies on using vertices in a clique that are not connected to any vertices not in the clique, but these are rare in the test graphs. This will cause Leaf to behave very similarly to NeighbourCull, as it falls back on the removal of the vertex with the most neighbours. Hence, the results for Leaf and NeighbourCull are very similar.

### Leaf Protein Culling Server

We have implemented the Leaf method to provide datasets (http://www.bioinf.manchester.ac.uk/leaf/ or https://github.com/SimonCB765/Leaf). The website uses Leaf to cull subsets of the PDB or submitted user sequences. Pre-computed sets of non-redundant PDB chains can also be downloaded, along with the source code and data files needed to run the culling on a local machine. Pre-culled PDB datasets are available with various sequence identity cut offs, resolutions and R-value limits. Culled proteomes are available for *h. sapiens, E. coli, Arabidopsis thaliana, S. cerevisiae* and *M. musculus*.

## Discussion

When comparing algorithms, the one that clearly underperforms is GLP. This algorithm substantially underperforms when compared to Leaf, and occasionally when compared to PISCES. Even when the time was increased to 500 times that of Leaf, the datasets returned by GLP were still smaller than those returned by Leaf. GLP has to work on very large graphs as it uses the complement of the protein similarity graph. While this problem can be overcome by using large amounts of memory, the time needed to produce a suitable result on larger graphs is far too large. For these reasons it is undesirable to use GLP, at present, for starting sets of this nature.

The Leaf algorithm consistently outperformed the other algorithms on both the datasets of random proteins, and the datasets of biological importance. A larger set will increase the ability to reveal significant differences between sets that are not apparent with smaller sets and give more accurate statistics on the properties of the set. It is not a general solution to the MIS problem, however, as its relatively poor performance on the BHOSLIB Benchmark data sets, suggest that it is only appears suitable for sparse graphs.

### Conclusions

Using algorithms designed to find maximum independent sets can substantially increase the size of non-redundant sets of proteins. For a small datasets of up to 1000 proteins, Cliquer can find the exact MIS, though other algorithms often find it too. The long run time of Cliquer prohibits its use for larger sets, however. For larger sets, the novel method Leaf is the most suitable for finding non-redundant protein datasets of maximal size, as it finds the largest sets in a short time. For sets with many edges per node, such as the BHOSLIB benchmarks, Leaf is not suitable.

## Supporting Information

Figure S1 **Comparisons of Leaf and GLP algorithms to PISCES.**
(DOCX)

Figure S2 **Cumulative Frequency Plot of Protein Lengths of Datasets from Human Proteome from Leaf and PISCES Algorithms Compared to Entire Proteome.**
(DOCX)

Table S1 **Algorithm Performances on Model Organisms.**
(XLSX)

Table S2 **Results from Culling PDB Data Sets.**
(DOCX)

## References

[1] Barton GJ (1996) Protein sequence alignment and database scanning. in: MJE Sternberg (ed.) *Protein Structure Prediction*. Oxford University Press, Oxford, 1996; 31 pp.

[2] Altschul SF, Gish W, Miller W, Myers EW, Lipman DJ (1990) Basic local alignment search tool. J. Mol. Biol. 215: 403–410. 10.1016/S0022-2836(05)80360-2 2231712

[3] Altschul SF, Madden TL, Scha?ffer AA, Zhang J, Zhang Z, et al (1997) Gapped BLAST and PSI-BLAST: A new generation of protein database search programs. Nuc. Acids Res. 25: 3389–3402. 10.1093/nar/25.17.3389 PMC146917 9254694

[4] Liu P, Zeng Z, Qian Z, Feng K, Cai Y (2009) Bioinformatics and Biomedical Engineering 1–3.

[5] Wang GL, Dunbrack RL (2003) PISCES: a protein sequence culling server. Bioinformatics 19: 1589–1591. 12912846 10.1093/bioinformatics/btg224

[6] Hobohm U, Sander C (1994) Enlarged representative set of protein structures. Prot. Sci. 3: 522–524. 10.1002/pro.5560030317 PMC2142698 8019422

[7] Grosso A, Locatelli M, Pullan W (2008) Simple ingredients leading to very efficient heuristics for the maximum clique problem. J. Heuristics 14: 587–612.

[8] Balaji S, Swaminathan V, Kannan K (2010) A simple algorithm to optimize maximum independent set. Adv. Modeling Optimization 12: 107–118.

[9] Östergård PRJ (2002) A fast algorithm for the maximum clique problem. Discrete Applied Mathematics 120: 197–207.

[10] Niskanen S, Östergård PRJ (2003) *Cliquer User's Guide, Version 1.0*; Communications Laboratory, Helsinki University of Technology.

[11] Thain D, Tannenbaum T, Livny M (2005) Distributed computing in practice: the Condor experience. Concurrency - Practice and Experience 17: 323–356.

