# Supplementary material for: Maximising the Size of Non-Redundant Protein Datasets Using Graph Theory
Source: PLoS One. 2013 Feb 5;8(2):e55484. doi: 10.1371/journal.pone.0055484 (PMC3564766; doi:10.1371/journal.pone.0055484)
Supplement: Figure S1 — Comparisons of Leaf and GLP algorithms to PISCES. (DOCX) [file pone.0055484.s001.docx]

#### Supplementary Information 1 Comparisons of Leaf and GLP algorithms to PISCES

#### Maximising the Size of Non-Redundant Protein Data Sets Using Graph Theory

#### Simon C. Bull, Mark R. Muldoon and Andrew J. Doig

(f)

(e)

(d)

(c)

(b)

(a)

The percentage improvement over PISCES shown by the Leaf and GLP algorithms. Graph (a) is for the datasets of 100 proteins, (b) for the datasets of 250 proteins, (c) for the datasets of 500 proteins, (d) for the datasets of 1000 proteins, (e) for the datasets of 2000 proteins and (f) for the datasets of 5000 proteins.
