# Supplementary material for: Maximising the Size of Non-Redundant Protein Datasets Using Graph Theory
Source: PLoS One. 2013 Feb 5;8(2):e55484. doi: 10.1371/journal.pone.0055484 (PMC3564766; doi:10.1371/journal.pone.0055484)
Supplement: Figure S2 — Cumulative Frequency Plot of Protein Lengths of Datasets from Human Proteome from Leaf and PISCES Algorithms Compared to Entire Proteome. (DOCX) [file pone.0055484.s002.docx]

#### Supplementary Information 2 Cumulative Frequency Plot of Protein Lengths of Datasets from Human Proteome from Leaf and PISCES Algorithms Compared to Entire Proteome.

#### Maximising the Size of Non-Redundant Protein Data Sets Using Graph Theory

#### Simon C. Bull, Mark R. Muldoon and Andrew J. Doig
