# Supplementary material for: Maximising the Size of Non-Redundant Protein Datasets Using Graph Theory
Source: PLoS One. 2013 Feb 5;8(2):e55484. doi: 10.1371/journal.pone.0055484 (PMC3564766; doi:10.1371/journal.pone.0055484)
Supplement: Table S2 — Results from Culling PDB Data Sets. (DOCX) [file pone.0055484.s004.docx]

#### Supplementary Information 4 Results from Culling PDB Data Sets

#### Maximising the Size of Non-Redundant Protein Data Sets Using Graph Theory

#### Simon C. Bull, Mark R. Muldoon and Andrew J. Doig

| % Maximum Sequence Identity | Minimum Resolution | Maximum R-Factor | #Proteins from PISCES | #Proteins from Leaf | % Improvement |
| --- | --- | --- | --- | --- | --- |
| 20 | 1.6 | 0.25 | 1886 | 2021 | 7.2 |
| 20 | 1.8 | 0.25 | 2954 | 3214 | 8.8 |
| 20 | 2.0 | 0.25 | 4030 | 4459 | 10.6 |
| 20 | 2.2 | 1.0 | 4640 | 5179 | 11.6 |
| 20 | 2.5 | 1.0 | 5346 | 5962 | 11.5 |
| 20 | 3.0 | 1.0 | 5922 | 6577 | 11.1 |
| 25 | 1.6 | 0.25 | 2276 | 2415 | 6.1 |
| 25 | 1.8 | 0.25 | 3677 | 3967 | 7.9 |
| 25 | 2.0 | 0.25 | 5089 | 5570 | 9.5 |
| 25 | 2.2 | 1.0 | 5910 | 6518 | 10.3 |
| 25 | 2.5 | 1.0 | 6822 | 7569 | 10.9 |
| 25 | 3.0 | 1.0 | 7525 | 8367 | 11.2 |
| 30 | 1.6 | 0.25 | 2676 | 2772 | 3.6 |
| 30 | 1.8 | 0.25 | 4469 | 4699 | 5.1 |
| 30 | 2.0 | 0.25 | 6360 | 6765 | 6.4 |
| 30 | 2.2 | 1.0 | 7492 | 7986 | 6.6 |
| 30 | 2.5 | 1.0 | 8713 | 9337 | 7.2 |
| 30 | 3.0 | 1.0 | 9615 | 10337 | 7.5 |
| 40 | 1.6 | 0.25 | 3182 | 3259 | 2.4 |
| 40 | 1.8 | 0.25 | 5604 | 5778 | 3.1 |
| 40 | 2.0 | 0.25 | 8337 | 8612 | 3.3 |
| 40 | 2.2 | 1.0 | 10029 | 10392 | 3.6 |
| 40 | 2.5 | 1.0 | 11872 | 12338 | 3.9 |
| 40 | 3.0 | 1.0 | 13219 | 13762 | 4.1 |
| 50 | 1.6 | 0.25 | 3524 | 3567 | 1.2 |
| 50 | 1.8 | 0.25 | 6308 | 6414 | 1.7 |
| 50 | 2.0 | 0.25 | 9571 | 9744 | 1.8 |
| 50 | 2.2 | 1.0 | 11657 | 11885 | 2.0 |
| 50 | 2.5 | 1.0 | 13897 | 14210 | 2.3 |
| 50 | 3.0 | 1.0 | 15584 | 15937 | 2.3 |
| 60 | 1.6 | 0.25 | 3704 | 3743 | 1.1 |
| 60 | 1.8 | 0.25 | 6737 | 6830 | 1.4 |
| 60 | 2.0 | 0.256 | 10336 | 10491 | 1.5 |
| 60 | 2.2 | 1.0 | 12679 | 12865 | 1.5 |
| 60 | 2.5 | 1.0 | 15249 | 15502 | 1.7 |
| 60 | 3.0 | 1.0 | 17221 | 17523 | 1.8 |
| 70 | 1.6 | 0.25 | 3845 | 3874 | 0.8 |
| 70 | 1.8 | 0.25 | 7069 | 7136 | 0.9 |
| 70 | 2.0 | 0.257 | 10927 | 11046 | 1.1 |
| 70 | 2.2 | 1.0 | 13469 | 13625 | 1.2 |
| 70 | 2.5 | 1.0 | 16282 | 16502 | 1.4 |
| 70 | 3.0 | 1.0 | 18463 | 18727 | 1.4 |
| 80 | 1.6 | 0.25 | 3979 | 4000 | 0.5 |
| 80 | 1.8 | 0.25 | 7341 | 7401 | 0.8 |
| 80 | 2.0 | 0.256 | 11416 | 11535 | 1.0 |
| 80 | 2.2 | 1.0 | 14128 | 14288 | 1.1 |
| 80 | 2.5 | 1.0 | 17154 | 17382 | 1.3 |
| 80 | 3.0 | 1.0 | 19535 | 19828 | 1.5 |
| 90 | 1.6 | 0.25 | 4112 | 4132 | 0.5 |
| 90 | 1.8 | 0.25 | 7650 | 7716 | 0.9 |
| 90 | 2.0 | 0.25 | 12009 | 12156 | 1.2 |
| 90 | 2.2 | 1.0 | 14950 | 15133 | 1.2 |
| 90 | 2.5 | 1.0 | 18250 | 18501 | 1.4 |
| 90 | 3.0 | 1.0 | 20916 | 21213 | 1.4 |
